# *cypress*: an R/Bioconductor package for cell-type-specific differential expression analysis power assessment

**DOI:** 10.1093/bioinformatics/btae511

**Published:** 2024-08-17

**Authors:** Shilin Yu, Guanqun Meng, Wen Tang, Wenjing Ma, Rui Wang, Xiongwei Zhu, Xiaobo Sun, Hao Feng

**Affiliations:** Department of Quantitative Health Sciences, Lerner Research Institute, Cleveland Clinic Foundation, Cleveland, OH 44106, United States; Department of Population and Quantitative Health Sciences, Case Western Reserve University, Cleveland, OH 44106, United States; Department of Population and Quantitative Health Sciences, Case Western Reserve University, Cleveland, OH 44106, United States; Department of Biostatistics, University of Michigan, Ann Arbor, MI 48109, United States; Department of Surgery, Case Western Reserve University, Cleveland, OH 44106, United States; Division of Surgical Oncology, Department of Surgery, University Hospitals Cleveland Medical Center, Cleveland, OH 44106, United States; Department of Pathology, Case Western Reserve University, Cleveland, OH 44106, United States; School of Statistics and Mathematics, Zhongnan University of Economics and Law, Wuhan, Hubei 430073, China; Department of Population and Quantitative Health Sciences, Case Western Reserve University, Cleveland, OH 44106, United States

## Abstract

**Summary:**

Recent methodology advances in computational signal deconvolution have enabled bulk transcriptome data analysis at a finer cell-type level. Through deconvolution, identifying cell-type-specific differentially expressed (csDE) genes is drawing increasing attention in clinical applications. However, researchers still face a number of difficulties in adopting csDE genes detection methods in practice, especially in their experimental design. Here we present *cypress*, the first experimental design and statistical power analysis tool in csDE genes identification. This tool can reliably model purified cell-type-specific (CTS) profiles, cell-type compositions, biological and technical variations, offering a high-fidelity simulator for bulk RNA-seq convolution and deconvolution. *cypress* conducts simulation and evaluates the impact of multiple influencing factors, by various statistical metrics, to help researchers optimize experimental design and conduct power analysis.

**Availability and implementation:**

*cypress* is an open-source R/Bioconductor package at https://bioconductor.org/packages/cypress/.

## 1 Introduction

Bulk RNA-sequencing technology has now been routinely adopted in clinical studies to investigate transcriptome profile alterations associated with phenotypes-of-interest. For example, researchers can use bulk RNA-sequencing to quantify and compare gene expression profiles between cancer subjects and healthy controls to identify Differentially Expressed (DE) genes (Wang *et al.* 2019a, Craig *et al.* 2021, Ergin *et al.* 2022). Meanwhile, real clinical samples often contain a mixture of different cell types; thus, the real clinical transcriptome signals are mosaics of signals from pure cell types. As a result, the bulk data we observed are the weighted average of signals from multiple cell types. In the past several years, computational deconvolution methods were developed to solve for cell-type mixture proportions. More recently, identifying cell-type-specific Differentially Expressed (csDE) genes has been made possible by methodology extensions of cell-type deconvolution (Li *et al.* 2019, Rahmani *et al.* 2019, Jin *et al.* 2021).

Although csDE genes calling is a more appealing and precise approach to detect alterations at cell-type resolution, clinical researchers still face a number of difficulties in using csDE detection methods in practice. First, determining the sample size in study participants recruiting is an important experimental design decision and is required by almost all studies and grant applications. Methods for sample size calculation in the context of csDE calling are not yet available. Second, unlike traditional hypothesis testing, where the testing performance can be easily predetermined, csDE detection’s sensitivity depends on various factors, including proportions of cell types, expression levels, sequencing depth, and biological dispersion among replicates. A rigorous experimental design requires the statistical power assessment method that considers these factors. Currently, however, such a method is yet to be developed.

Here, we present a statistical framework and tool *cypress* (cell-type-specific differential expression power assessment) to guide experimental design, assess statistical power, and optimize sample size requirements for csDE analysis. In this framework, we (i) reliably model purified CTS profiles, cell-type compositions, and biological and technical variations; (ii) provide thorough assessment using multiple statistical metrics; (iii) conduct extensive simulation studies using parameters estimated from real data; and (iv) provide a user-friendly tool to researchers for their own data simulation and power evaluation. To the best of our knowledge, *cypress* is the first power assessment tool available for csDE analysis using bulk sequencing data alone.

## 2 Methods

### 2.1 Statistical framework behind *cypress*

**Figure 1.**
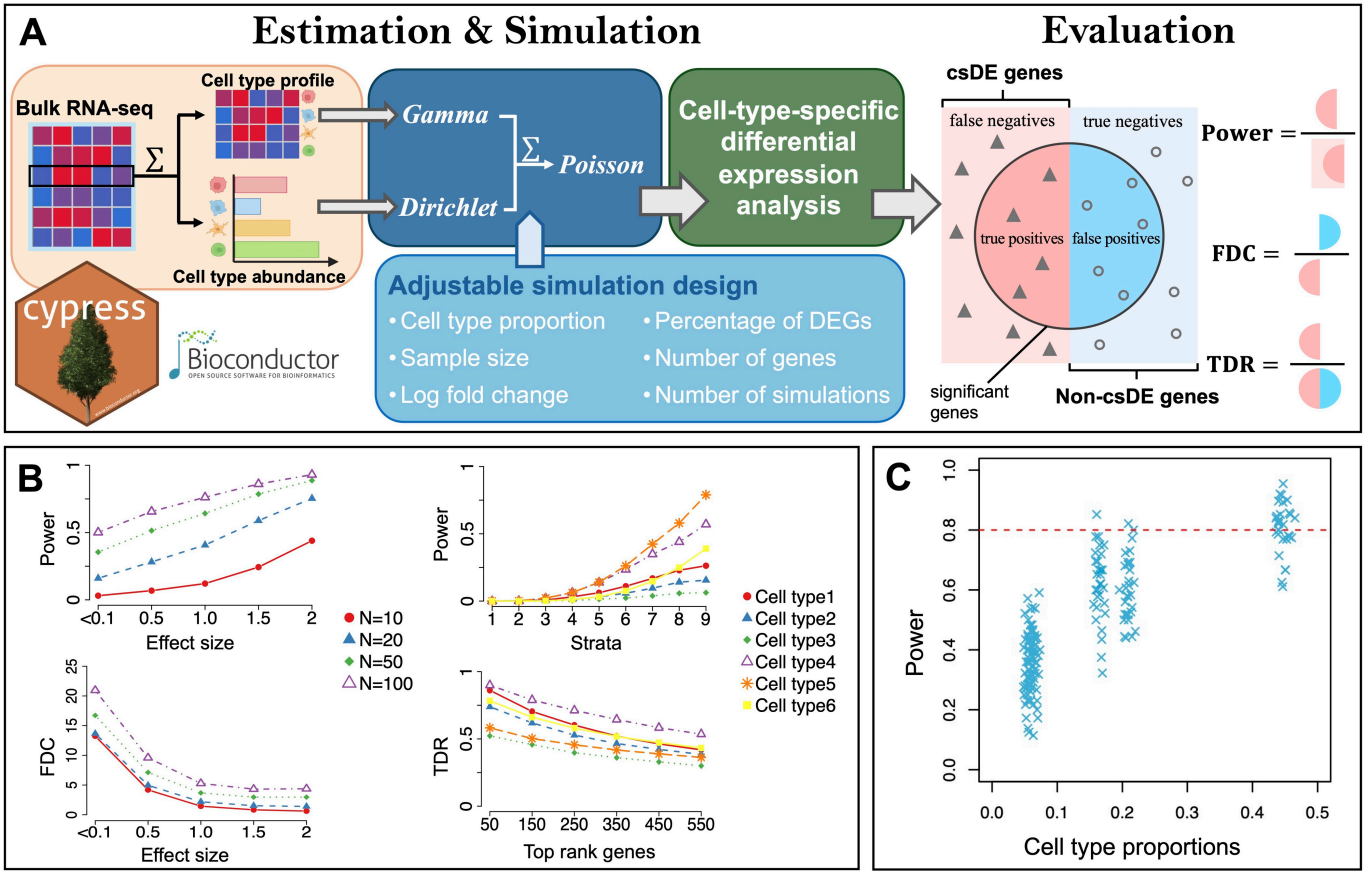
Schematic overview of *cypress* and evaluation metrics. (A) The bulk RNA-seq data for each sample can be deconvolved into purified CTS expression profiles and their associated weights. These two components are modeled by *Gamma* and *Dirichlet* distributions, respectively. Adjusting distribution parameters enables the inspection of the impact of various factors in the experimental design of CTS differential expression studies. Several evaluation metrics are provided, including statistical power, FDC, and TDR. (B) Applications of *cypress* to study influencing factors in experimental design by real cell line sequencing data-based simulation. Metrics can be decomposed for each cell type. (C) Impact of cell type abundance on statistical power in one scenario with a sample size of 50 and an effect size of 0.5. Cell type proportions are simulated from a *Dirichlet* distribution.

An essential step in the framework of *cypress* is to reliably characterize the cell type mixing process, i.e. the mixed data generative model. In agreement with previous studies (Newman *et al.* 2015, Wang *et al.* 2019b, Jin *et al.* 2021, Feng *et al.* 2023, Meng *et al.* 2023), we designed a framework to combine *Gamma*, *Dirichlet*, and *Poisson* distributions into the convolution process. An overview of this framework is illustrated in Fig. 1A. The purified CTS profile (subject to a log transformation) can be modeled by a *Gamma* distribution. This allows us to effectively capture the biological variations among different phenotypic groups, as well as the dispersions inherent in the underlying CTS gene expression. Naturally, *Dirichlet* distribution is adopted to model cell-type compositions. The summation of the *Dirichlet* parameters would control the composition variability across samples. After a weighted average of the profiles, where the weights are from *Dirichlet* and the profiles are from *Gamma*, the admixed outcomes are fed into *Poisson* distribution to simulate technical variations from sequencing experiments. This framework enables controlling and adjusting mean and dispersion from each source, thus allowing us to assess their impact later. The detailed statistical framework is illustrated in Supplementary Material.

### 2.2 Evaluation metrics in power analysis

We adopted several key evaluation metrics in the power analysis of csDE testing: statistical power, true discovery rate (TDR), and false discovery cost (FDC), as explained visually in Fig. 1A. *cypress* assumes DE gene is present in only one cell type for each gene. Statistical power inspects the sensitivity of genome-wide testing to determine if one could correctly reject the null when the underlying is true csDE. TDR is defined as the counterpart of false discovery rate (FDR): TDR = 1–FDR. After sorting genes by their significance, TDR can be calculated at any arbitrary top-ranking cutoff. It reflects the pragmatic performance of a testing method where the researchers focus on the top identified features/genes; thus, the fidelity among them matters the most. We also introduced a new metric named FDC, which is defined as the ratio of the number of false positives over the number of true positives. It naturally reflects the expected “cost” of false discoveries for each true discovery we make.

We reported these metrics at various gene expression log-fold-change levels (i.e. effect sizes) and various sample sizes, as illustrated in the left two figures of Fig. 1B. Results were presented after stratifying genes into several different strata based on their gene expression levels from low to high, as depicted in the right two figures of Fig. 1B, as it had been shown to help a thorough power inspection (Chhangawala *et al.* 2015, Wu *et al.* 2015, Feng *et al.* 2023, Meng *et al.* 2023). Note that the above-mentioned metrics were reported for each cell type, naturally, as each cell type has its own power profile in csDE analysis. We also inspected the associations between these metrics and cell type abundance (Fig. 1C).

### 2.3 Implementation and user options

*cypress* was developed as an R/Bioconductor software package that is publicly available, with a package vignette containing detailed tutorials. The default csDE genes identification algorithm we adopted here is TOAST, as it has been shown to be a leading method in csDE genes calling (Li *et al.* 2019, Meng *et al.* 2023). Alternatively, users could choose CeDAR (Chen *et al.* 2023) or DESeq2 (Love *et al.* 2014). In practical usage, *cypress* provides three different options, simFromData(), simFromParam(), and quickPower(), for users to conduct experimental design and power analysis. First, it allows users to provide their own pilot bulk RNA-seq data for csDE genes detection and to run simulations based on parameters estimated from this data. If cell type proportions are unavailable, *cypress* employs a Reference-Free (RF) deconvolution algorithm, *RefFreeEWAS* (Houseman *et al.* 2016). However, Reference-Based (RB) methods, such as CIBERSORT (Newman *et al.* 2015), MuSiC (Wang *et al.* 2019b), and imply (Meng *et al.* 2024), are recommended for their superior accuracies (Avila Cobos *et al.* 2020). Users can choose any top-tier RB methods and feed the cell type proportions to *cypress*. Second, for experimental designs without pilot data, users have the option to borrow one of our pre-estimated parameter sets from three real datasets (Haberman *et al.* 2014, Linsley *et al.* 2014, Parikshak *et al.* 2016, Gandal *et al.* 2018, Loberman-Nachum *et al.* 2019) and conduct simulations and evaluations under their designated scenarios. Lastly, if no prior information is provided at all, *cypress* has a quickpower() function to directly print out pre-evaluated outcomes based on our saved simulations from real datasets. This provides a quick reference for researchers without historical bulk data to deconvolve and leverage on. Overall, it can serve the needs of researchers who want to conduct experimental design, with or without pilot data. *cypress* includes integrated plotting functions such as plotPower(), plotTDR(), and plotFDC(). Detailed software usage is in the package’s vignette.

## 3 Application results

We used datasets from a sequencing experiment with multiple pure cell lines (Linsley *et al.* 2014), estimated cell type profiles, and conducted simulations with *cypress* to study impact factors in csDE analysis. A total of 30 simulations were conducted in each scenario, and the results were averaged. Key differences between cell types are provided in the Supplementary Material. As shown in Fig. 1B, the top-left figure shows the relationship between statistical power and effect size, at various sample sizes. As expected, the power increases as the effect size increases. We would enjoy a more drastic power gain as the sample size increases when the sample size is small (i.e. 10 or 20 per group). However, such gain effects would diminish when we already have a moderate sample size (i.e. 50). The effect size plays a significant role in the “cost” of csDE testing, as indicated by the bottom-left panel in Fig. 1B. The FDC would drop drastically as the effect size increases, which indicates a reduced number of false positives per true positive we obtain. Stratified power analyses were also conducted based on the underlying gene expression levels. Here, stratum 1–9 were introduced: [0, 10], [10, 20], [20, 40], [40, 80], [80, 160], [160, 320], [320, 640], [640, 1280], and [1280, + *∞*]; power analyses were conducted at each stratum respectively. These cutoffs are empirical and help us explore key performance trends as gene expression increases. Results shown in the top-right panel of Fig. 1B indicate deficient statistical power among low strata. This is because low baseline expression levels and shallow sequencing depths are overwhelmed by technical noise, complicating csDE testing. The TDR plot on the bottom-right shows the accuracy of top-ranking genes called to be significant csDE genes. In general, the true positive rate would decrease as we move down on the significance ranking list. As a considerable number of biomedical researchers would only use the csDE genes detection method as a biomarker discovery tool, the accuracy of biomarkers detection is crucial among top-ranking ones. TDR plot gives users practical guidance for accuracy should one only want to inspect the top-ranking significant biomarkers and follow up with those. Cell type abundance is also positively associated with the statistical power for csDE gene detection within that cell type. Results in Fig. 1C and Supplementary Fig. S1 show that as a cell type increases its abundance, its statistical power would also increase. In practice, researchers could expect higher power for more abundant cell types in a study. Note the above metrics can be decomposed for each cell type, as seen in Fig. 1B and in Supplementary Figs S2–S6. Similar conclusions are observed at various numbers of genes and simulations, and *cypress* can provide comprehensive evaluations within minutes (Supplementary Tables S4 and S6).

## 4 Conclusion

As researchers are increasingly aware that the observed signal is a weighted average of pure cell type profiles in clinical bulk sequencing data, there is growing interest in adopting csDE genes detection methods in bulk data analysis. Our proposed tool, *cypress*, is the first available tool to serve the researcher’s needs to rigorously conduct experimental design to ensure the study is well-powered. *cypress* is capable of modeling and simulating various sources of variation in signal convolution and deconvolution and adopting multi-faceted statistical evaluation metrics in csDE genes hypothesis testing evaluation. It has high flexibility to take different types of input, regardless of the availability of pilot data.

## Supplementary Material

btae511_Supplementary_Data

## Data Availability

The *cypress* R/Bioconductor package is available at https://bioconductor.org/packages/cypress/.

## References

[btae511-B1] Avila Cobos F , Alquicira-Hernandez J, Powell JE et al Benchmarking of cell type deconvolution pipelines for transcriptomics data. Nat Commun 2020;11:5650. 33159064 10.1038/s41467-020-19015-1 PMC7648640

[btae511-B2] Chen L , Li Z, Wu H. CeDAR: incorporating cell type hierarchy improves cell type-specific differential analyses in bulk omics data. Genome Biol 2023;24:37. 36855165 10.1186/s13059-023-02857-5 PMC9972684

[btae511-B3] Chhangawala S , Rudy G, Mason CE et al The impact of read length on quantification of differentially expressed genes and splice junction detection. Genome Biol 2015;16:131–10. 26100517 10.1186/s13059-015-0697-y PMC4531809

[btae511-B4] Craig DW , Hutchins E, Violich I et al; Parkinson Progression Marker Initiative. RNA sequencing of whole blood reveals early alterations in immune cells and gene expression in Parkinson’s disease. Nature Aging 2021;1:734–47. 37117765 10.1038/s43587-021-00088-6

[btae511-B5] Ergin S , Kherad N, Alagoz M. RNA sequencing and its applications in cancer and rare diseases. Mol Biol Rep 2022;49:2325–33. 34988891 10.1007/s11033-021-06963-0 PMC8731134

[btae511-B6] Feng H , Meng G, Lin T et al ISLET: individual-specific reference panel recovery improves cell-type-specific inference. Genome Biol 2023;24:174. 37496087 10.1186/s13059-023-03014-8 PMC10373385

[btae511-B7] Gandal MJ , Zhang P, Hadjimichael E et al; PsychENCODE Consortium. Transcriptome-wide isoform-level dysregulation in ASD, schizophrenia, and bipolar disorder. Science 2018;362:eaat8127. 30545856 10.1126/science.aat8127 PMC6443102

[btae511-B8] Haberman Y , Tickle TL, Dexheimer PJ et al Pediatric Crohn disease patients exhibit specific ileal transcriptome and microbiome signature. J Clin Invest 2014;124:3617–33. 25003194 10.1172/JCI75436 PMC4109533

[btae511-B9] Houseman EA , Kile ML, Christiani DC et al Reference-free deconvolution of DNA methylation data and mediation by cell composition effects. BMC Bioinformatics 2016;17:259–15. 27358049 10.1186/s12859-016-1140-4 PMC4928286

[btae511-B10] Jin C , Chen M, Lin D-Y et al Cell-type-aware analysis of RNA-seq data. Nat Comput Sci 2021;1:253–61. 34957416 10.1038/s43588-021-00055-6 PMC8697413

[btae511-B11] Li Z , Wu Z, Jin P et al Dissecting differential signals in high-throughput data from complex tissues. Bioinformatics 2019;35:3898–905. 30903684 10.1093/bioinformatics/btz196 PMC6931351

[btae511-B12] Linsley PS , Speake C, Whalen E et al Copy number loss of the interferon gene cluster in melanomas is linked to reduced T cell infiltrate and poor patient prognosis. PLoS One 2014;9:e109760. 25314013 10.1371/journal.pone.0109760 PMC4196925

[btae511-B13] Loberman-Nachum N , Sosnovski K, Di Segni A et al Defining the celiac disease transcriptome using clinical pathology specimens reveals biologic pathways and supports diagnosis. Sci Rep 2019;9:16163. 31700112 10.1038/s41598-019-52733-1 PMC6838157

[btae511-B14] Love M , Anders S, Huber W. Differential analysis of count data—the deseq2 package. Genome Biol 2014;15:550. 25516281 10.1186/s13059-014-0550-8 PMC4302049

[btae511-B15] Meng G , Tang W, Huang E et al A comprehensive assessment of cell type-specific differential expression methods in bulk data. Brief Bioinform 2023;24:bbac516. 36472568 10.1093/bib/bbac516 PMC9851321

[btae511-B16] Meng G , Pan Y, Tang W et al imply: improving cell-type deconvolution accuracy using personalized reference profiles. Genome Med 2024;16:65. 38685057 10.1186/s13073-024-01338-z PMC11057104

[btae511-B17] Newman AM , Liu CL, Green MR et al Robust enumeration of cell subsets from tissue expression profiles. Nat Methods 2015;12:453–7. 25822800 10.1038/nmeth.3337 PMC4739640

[btae511-B18] Parikshak NN , Swarup V, Belgard TG et al Genome-wide changes in lncRNA, splicing, and regional gene expression patterns in autism. Nature 2016;540:423–7. 27919067 10.1038/nature20612 PMC7102905

[btae511-B19] Rahmani E , Schweiger R, Rhead B et al Cell-type-specific resolution epigenetics without the need for cell sorting or single-cell biology. Nat Commun 2019;10:3417. 31366909 10.1038/s41467-019-11052-9 PMC6668473

[btae511-B20] Wang J , Dean DC, Hornicek FJ et al RNA sequencing (RNA-seq) and its application in ovarian cancer. Gynecol Oncol2019a;152:194–201. 30297273 10.1016/j.ygyno.2018.10.002

[btae511-B21] Wang X , Park J, Susztak K et al Bulk tissue cell type deconvolution with multi-subject single-cell expression reference. Nat Commun2019b;10:380. 30670690 10.1038/s41467-018-08023-x PMC6342984

[btae511-B22] Wu H , Wang C, Wu Z. PROPER: comprehensive power evaluation for differential expression using RNA-seq. Bioinformatics 2015;31:233–41. 25273110 10.1093/bioinformatics/btu640 PMC4287952

