# Supplementary material for: cypress: an R/Bioconductor package for cell-type-specific differential expression analysis power assessment
Source: Bioinformatics. 2024 Aug 17;40(8):btae511. doi: 10.1093/bioinformatics/btae511 (PMC11357793; doi:10.1093/bioinformatics/btae511)
Supplement: btae511_Supplementary_Data [file btae511_supplementary_data.pdf]

cypress: an R/Bioconductor package for cell-type-specific differential expression analysis power assessment.

Supplementary Materials

Shilin Yu†, Guanqun Meng†, Wen Tang, Wenjing Ma, Rui Wang,
Xiongwei Zhu, Xiaobo Sun, Hao Feng*

Contents

1 Simulation details **2**
1.1 Gamma-Poisson compound 2
1.2 Cell-type specific profile generation 2
1.3 Cell-type proportion generation 3
1.4 Distinguish each cell type 3
1.5 RNA-seq count data 4

2 Simulation results **5**

3 Impact of the number of cell types **11**

4 Impact of the number of genetic features and simulations **12**

5 Impact of the initial reference-based deconvolution **13**

6 Impact of the estimated cell-type-specific expression matrices **14**

7 Impact of DE genes proportions **15**

8 Simulated data evaluations **16**

9 *cypress* Run time **18**

1 Simulation details

1.1 Gamma-Poisson compound

Using *Negative Binomial (NB)* distribution is a common practice to model RNA-seq data [Wu et al., 2015, Meng et al., 2023]. Given its equivalence to the *Gamma-Poisson* hierarchical distribution (shown below), RNA-seq data simulation can also be constructed from these two distributions for the sake of cell-type proportions incorporation:

$$Y \sim Poisson(\lambda)$$

$$\lambda \sim Gamma(shape = a, scale = b)$$

then Y is equivalent to a negative binomial distribution. For systematic details, please refer to [Meng et al., 2023].

Deconstructing the Negative Binomial (NB) model within the two-step $Gamma - Poisson$ process offers several advantages. It separates the control of the biological and technical noises respectively by the *Gamma* and *Poisson* distribution. Moreover, it facilitates the integration of cell-type proportion information into the underlying expressions generated by the *Gamma* distribution. This divide-and-reintegrate process allows us to fine-tuning of the cell-type proportion parameters while retaining the control over the underlying cell-type specific gene expression.

1.2 Cell-type specific profile generation

Notations and Overview

The simulation of cell-type specific profiles is based on real datasets and the simulation procedures are slightly different depending on the availability of cell-type abundances. We denote g as gene index, ranging from 1 to G, and i as sample index, ranging from 1 to N. The mixing proportion for each sample i is represented by $\boldsymbol{\theta}_i = (\theta_{1i}, \theta_{2i} \ldots, \theta_{ki})$, where $\sum_k \theta_{ki} = 1$ satisfies the constraint, with k indicating cell-type index. Furthermore, z_i represents a subject-specific phenotypical group assignment, where $z_i = 1/0$ denotes cases and controls, respectively, for two-group comparisons.

The underlying cell-type-specific gene expression for each phenotypical group is denoted as a vector $\boldsymbol{x}^T_{g,z=1/0} = (x_{g,z=1/0,1}, \ldots, x_{g,z=1/0,k})$, while the underlying gene expression across cell types for each subject, m_{gi}, represents a weighted sum of cell-specific expressions evaluated by subject-specific cell types proportions. Subsequently, RNA-seq read count data, Y_{gi}, is generated from a *Poisson* distribution.

$$m_{gi} = \boldsymbol{x}_{g,z=1/0}\boldsymbol{\theta}_i^T$$
$$Y_{gi}|m_{gi} \sim Poisson(m_{gi})$$

Cell-type proportions unavailable

If users are unable to provide cell-type proportions and could only supply bulk RNA-seq data, *cypress* employs a *reference-free* deconvolution approach to infer cell-type proportions for each sample. This inference is achieved through a quadratic programming matrix decomposition algorithm [Houseman et al., 2016]. Following this initial step, sample- and cell-type-specific expression profiles can be estimated using the *tensor* function provided by the *TCA* package [Rahmani et al., 2019].

The parameters for cell-specific gene expression are inferred from sample- and cell-type-specific expression profiles. The cell-specific underlying expression parameters for cell k and gene g are estimated using the *PROPER package* [Wu et al., 2015]. This package assumes that the read counts follow a NB distribution with parameters μ_{gk} and ϕ_{gk}, representing the mean expression and biological dispersion for gene g and cell-type k in log-scale.

We further employ a *Multivariate Normal* distribution to model the gene expression mean and dispersion across the entire genome. Utilizing Maximum Likelihood Estimation (MLE), we also estimate the variance-covariance structure for

both ($\hat{\mathbf{\Sigma}}m$ and $\hat{\mathbf{\Sigma}}\phi$), respectively.

$Multivariate - Gaussian$ distribution is also used to simulate two matrices, representing gene expression mean and dispersion parameters of cell-specific gene expression, for each phenotypical group. The underlying cell-specific gene expression matrix is simulated using a re-parameterized *Gamma* distribution for each group.

$$\begin{aligned}
\boldsymbol{M}^z_{G\times K} &\sim MVN(\boldsymbol{\mu}_m, \hat{\mathbf{\Sigma}}_m)\\
\mathbf{\Phi}_{G\times K} &\sim MVN(\boldsymbol{\mu}_\phi, \hat{\mathbf{\Sigma}}_\phi)\\
\boldsymbol{X}^z_{G\times K} &\sim \Gamma\{shape = \frac{1}{\exp(\mathbf{\Phi}_{G\times K})}, scale = \exp(\boldsymbol{M}^z_{G\times K}) \cdot \exp(\mathbf{\Phi}_{G\times K})\}
\end{aligned}$$

We assume cases and controls share the identical dispersion parameters, $\mathbf{\Phi}_{\boldsymbol{G\times K}}$, but different $\boldsymbol{M}^{\boldsymbol{z}}_{\boldsymbol{G\times K}}$, where $z = 1/0$. LFCs are randomly drawn from $N(\mu_{LFC}, 0.5)$.

Cell-type proportions available

If users provide cell-type proportions, the *reference-free* deconvolution process becomes redundant and is skipped. *cypress* would directly infer sample- and cell-type specific expression profiles and estimate cell-type-specific underlying expression parameters. Similarly to the methodology described above, gene expression mean and dispersion parameters are simulated using a $Multivariate - Gaussian$ distribution. Subsequently, the underlying cell-type specific gene expression matrix is generated using a re-parameterized *Gamma* distribution for each group.

1.3 Cell-type proportion generation

The cell-type proportions are simulated using a *Dirichlet* distribution based on $\boldsymbol{\alpha}$ parameters, where $\boldsymbol{\alpha}^T_C$ and $\boldsymbol{\alpha}^T_D$ represent the parameters for controls and cases, respectively.

$$\begin{aligned}
Control : \boldsymbol{\theta}^C_i &\sim Dirichlet(\boldsymbol{\alpha}^T_C)\\
Case : \boldsymbol{\theta}^D_i &\sim Dirichlet(\boldsymbol{\alpha}^T_D)
\end{aligned}$$

$\boldsymbol{\alpha}^T_C$ and $\boldsymbol{\alpha}^T_D$ are solved by *dirichlet.mle* function (*sirt* package) [Robitzsch, 2022] from the available sample-specific cell-type abundance information obtained from the previous section.

1.4 Distinguish each cell type

As noted in the supplementary materials section 1.2, the cell type-specific expression parameters, mean (μ_{gk}) and dispersion (ϕ_{gk}), for cell type k and gene g are estimated using the PROPER package. For each gene g, the mean and dispersion across all cell types are represented as $\boldsymbol{\mu}_g = (\mu_{g1}, \ldots, \mu_{gK})^T$ and $\boldsymbol{\phi}_g = (\phi_{g1}, \ldots, \phi_{gK})^T$, respectively. To account for correlations among multiple cell types, we apply a *multivariate normal distribution* (MVN) to both $\boldsymbol{\mu}_g$ and $\boldsymbol{\phi}_g$:

$$\boldsymbol{\mu}_g \sim MVN(\boldsymbol{m}, \mathbf{\Sigma}_m), \quad \boldsymbol{\phi}_g \sim MVN(\boldsymbol{d}, \mathbf{\Sigma}_d)$$

Maximum likelihood estimation (MLE) is used to estimate the parameters $\boldsymbol{m}$, $\mathbf{\Sigma}_m$, $\boldsymbol{d}$, and $\mathbf{\Sigma}_d$, where $\boldsymbol{m}$ and $\boldsymbol{d}$ are vectors of mean values for the six cell types, and $\mathbf{\Sigma}_m$ and $\mathbf{\Sigma}_d$ are 6×6 variance-covariance matrices. This approach guarantees that each cell type is not only characterized by unique mean and dispersion parameters but also by distinct variance-covariance relationships with other cell types.

For example, in Figure 1B from the main manuscript, simulation parameters are derived from an immune-associated disease (IAD) study (GSE60424), which includes six cell types: B-Cell, CD4, CD8, Monocytes, Neutrophils, and NK. The estimated mean ($\hat{\boldsymbol{m}}$) and dispersion vectors ($\hat{\boldsymbol{d}}$), along with their respective variance-covariance matrices ($\hat{\mathbf{\Sigma}}_m$ and $\hat{\mathbf{\Sigma}}_d$) are

detailed in Table S1 and Table S2. These parameters capture the means and dispersions of the cell-type-specific expression matrix. In the simulation process, we use 'Cell type 1' through 'Cell type 6' as labels to mask the names of these cell types. As shown in Table S1 below, monocytes is the most abundant cell (4.72) and neutrophils has the largest dispersion (4.05). We can also observe that monocytes and neutrophils have strong correlation (2.03).

$\hat{\boldsymbol{m}}$, $\hat{\boldsymbol{\Sigma}}_m$	B-Cell	CD4	CD8	Monocytes	Neutrophils	NK
	4.53	4.48	4.56	4.72	4.49	4.53
B-cell	2.19	1.73	1.69	1.50	1.30	1.47
CD4		2.09	1.98	1.38	1.26	1.63
CD8			2.02	1.42	1.25	1.73
Monocytes				2.16	2.03	1.41
Neutrophils					4.05	1.44
NK						1.08

Table S1: Estimated mean vector ($\hat{\boldsymbol{m}}$) and variance-covariance matrix ($\hat{\boldsymbol{\Sigma}}_m$) for means of the cell-type-specific expression matrix.

$\hat{\boldsymbol{d}}$, $\hat{\boldsymbol{\Sigma}}_d$	B-Cell	CD4	CD8	Monocytes	Neutrophils	NK
	4.53	4.48	4.56	4.72	4.49	4.53
B-cell	2.19	1.73	1.69	1.50	1.30	1.47
CD4		2.09	1.98	1.38	1.26	1.63
CD8			2.02	1.42	1.25	1.73
Monocytes				2.16	2.03	1.41
Neutrophils					4.05	1.44
NK						1.08

Table S2: Estimated mean vector ($\hat{\boldsymbol{d}}$) and variance-covariance matrix ($\hat{\boldsymbol{\Sigma}}_d$) for dispersions of the cell-type-specific expression matrix.

1.5 RNA-seq count data

For each sample, we could obtain the underlying gene expression, m_{gi}, through a weighted sum of cell-specific gene expression, $\boldsymbol{x}_{gi}$, evaluated by $\boldsymbol{\theta}_i^T$. Conditioning on m_{gi}, RNA-seq count data are simulated by *Poisson* Distributions.

$$Y_{gi}|m_{gi} \sim Poisson(m_{gi})$$

2 Simulation results

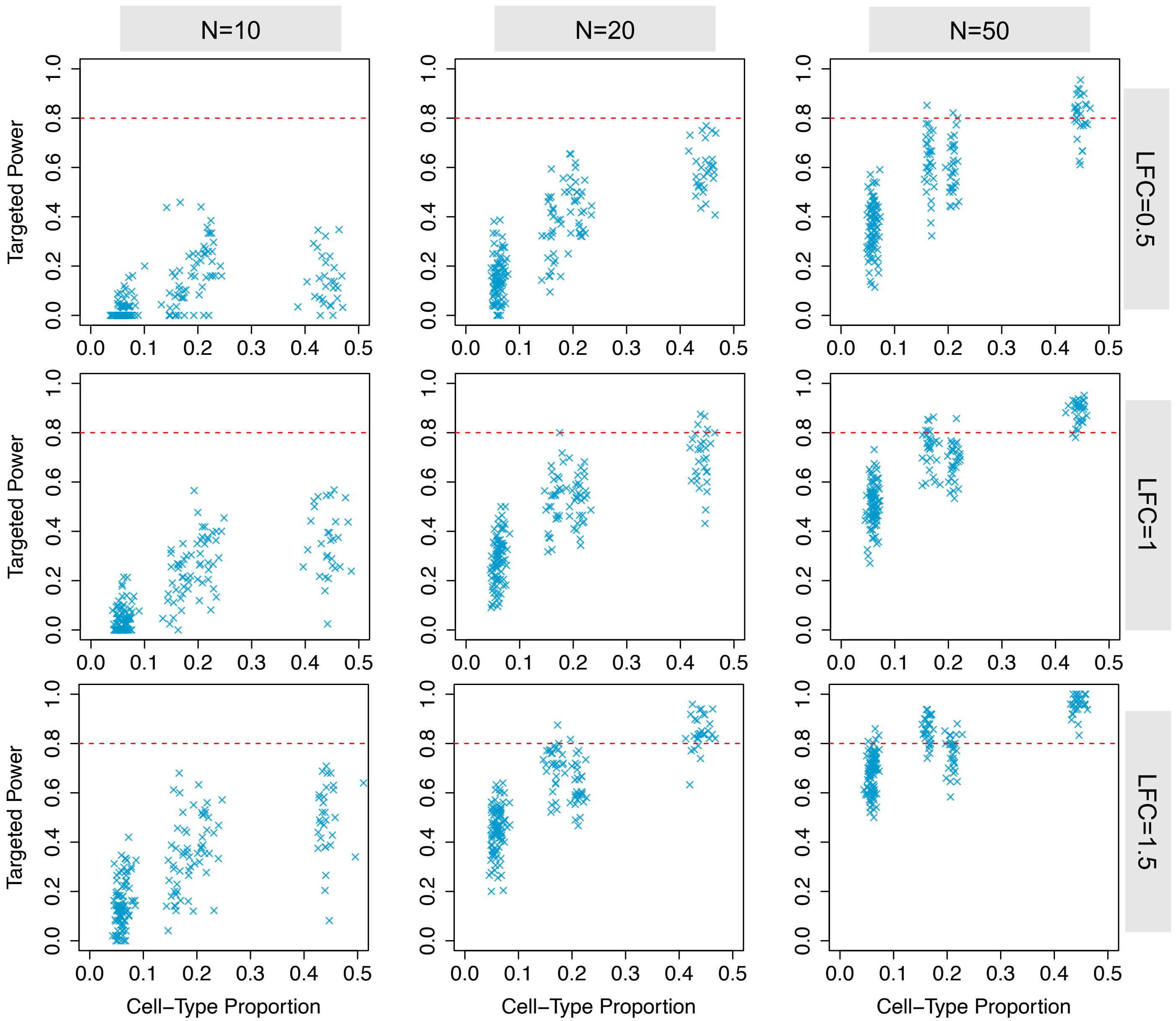

Figure S1: The figure illustrates the target power versus cell-type abundances under different simulation scenarios. Each row corresponds to a different effect size (LFC) of 0.5, 1, and 1.5, respectively, while each column represents a different sample size (N) of 10, 20, and 50, respectively. The target power threshold is LFC=0.5.

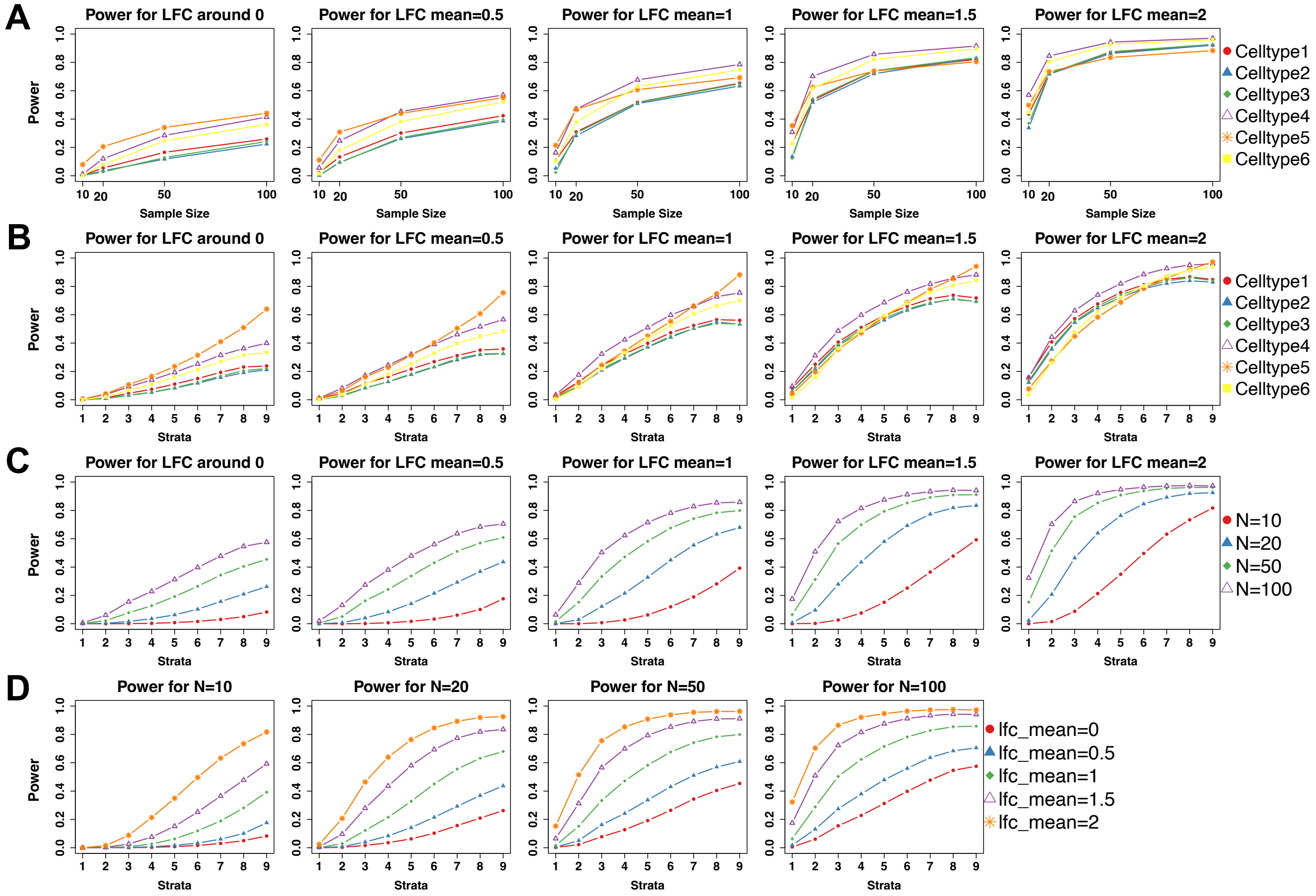

Figure S2: Statistical power influenced by sample size, effect size, and gene expression strata. Each point on the line plot is an average value over N=30 simulations based on a real bulk RNA-seq data with 6 cell lines (GSE60424). Log fold change (LFC) has a distribution with a mean given in each panel, with a standard deviation of 0.5. (A) Statistical power by sample size under the scenario of increasing log fold change mean value, each line represents one cell type. (B) Statistical power by strata under the scenario of increasing LFC mean value, each line represents one cell type. Statistical power was the average value across sample sizes. (C) Statistical power by strata under the scenario of increasing LFC mean value, each line represents one sample size. Statistical power was the average value across cell types. (D) Statistical power by strata under the scenario of increasing sample size, each line represents a LFC mean value. Statistical power was averaged across cell types.

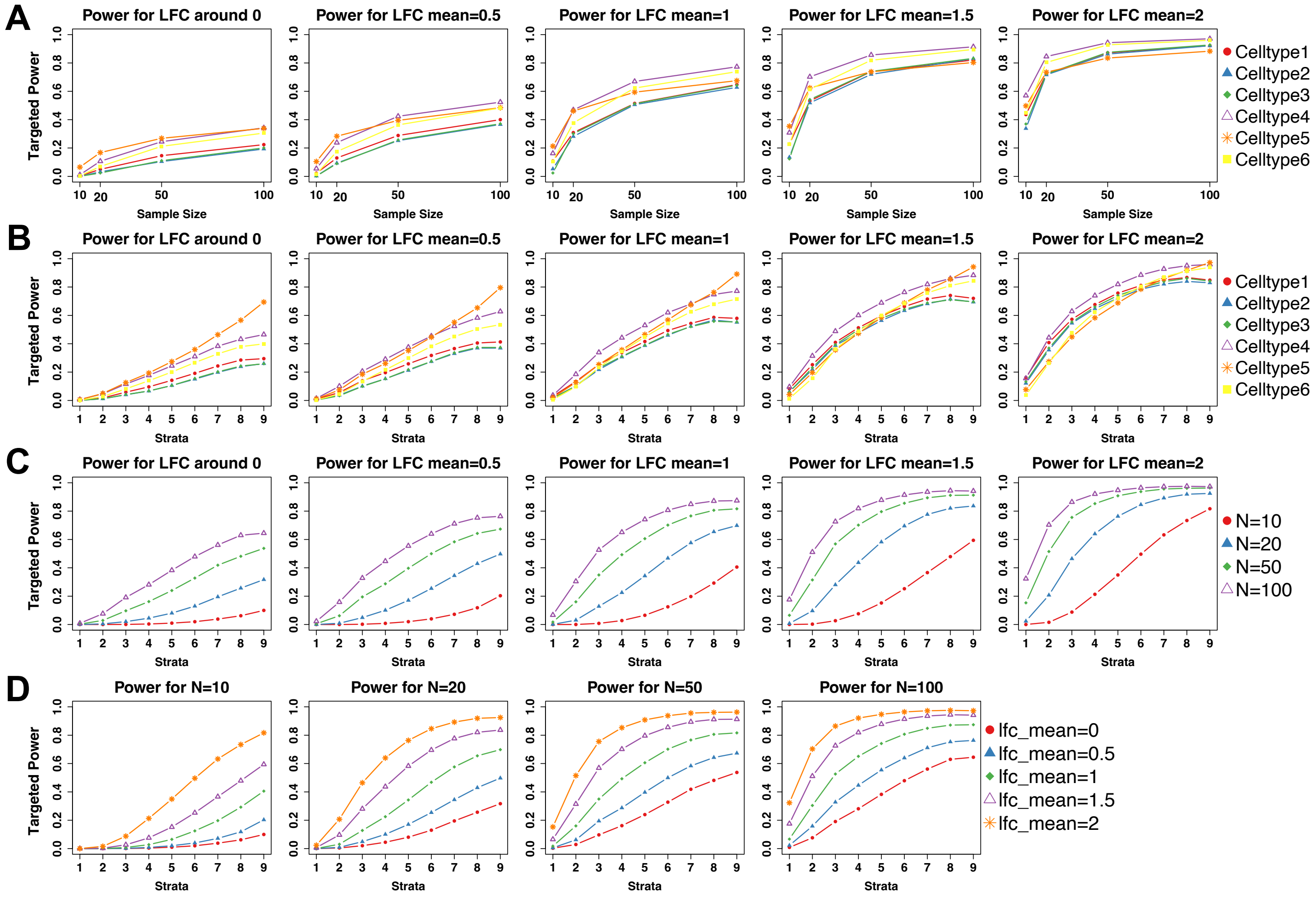

Figure S3: The targeted power using the threshold of 0.2 LFC as the qualifying criteria. The remaining descriptions of (A)-(D) are the same as in Figure S2.

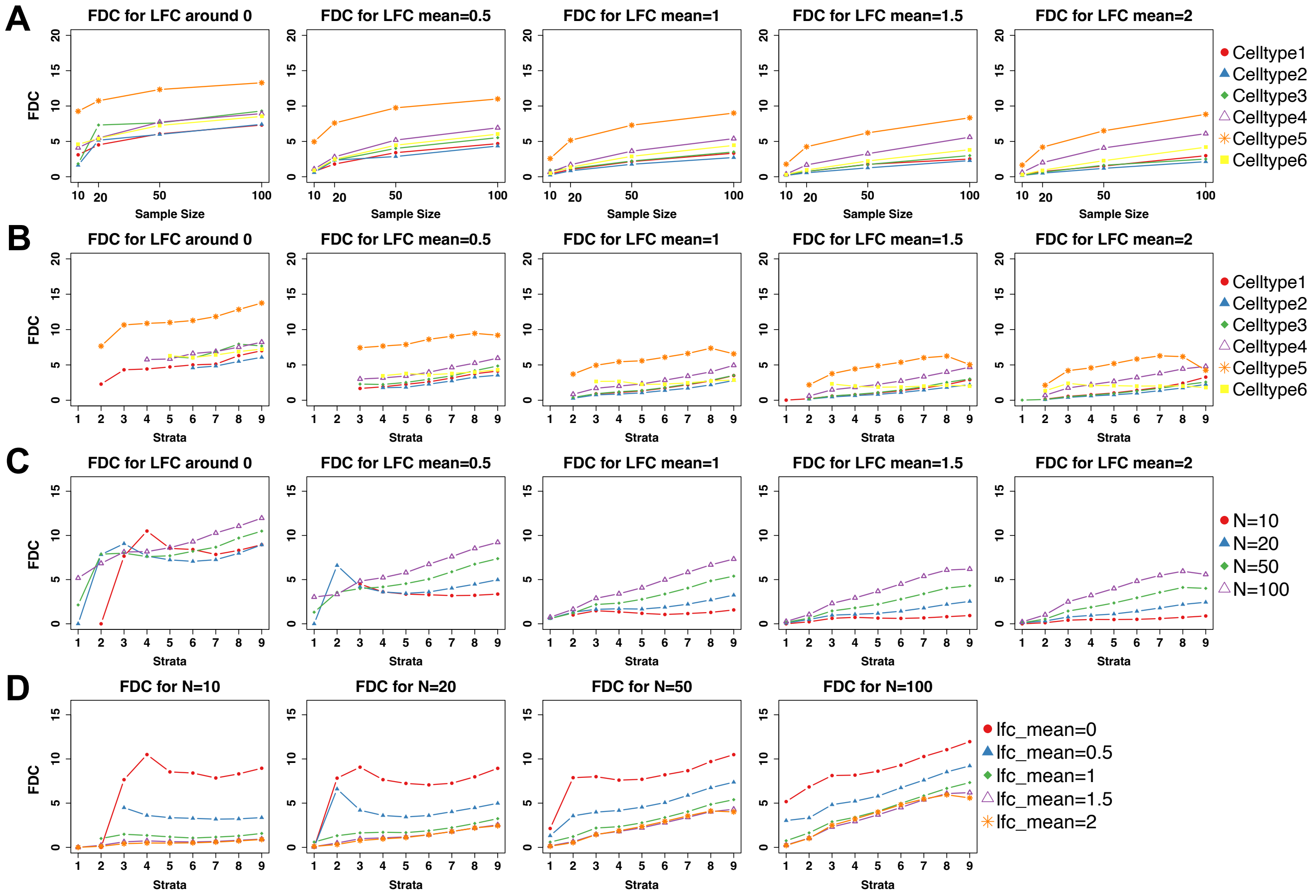

Figure S4: False discovery cost (FDC) influenced by sample size, effect size, and gene expression strata. Each point on the line plot is an average value over N=30 simulations based on a real bulk RNA-seq data with 6 cell lines (GSE60424). Log fold change (LFC) has a distribution with a mean given in each panel, with a standard deviation of 0.5. (A) False discovery cost by sample size under the scenario of increasing LFC mean value, each line represents one cell type. (B) False discovery cost by strata under the scenario of increasing LFC mean value, each line represents one cell type. FDC was the average value across sample sizes. (C) False discovery cost by strata under the scenario of increasing LFC mean value, each line represents one sample size. FDC was the average value across cell types. (D) False discovery cost by strata under the scenario of increasing sample size, each line represents LFC mean value. FDC was averaged across sample sizes.

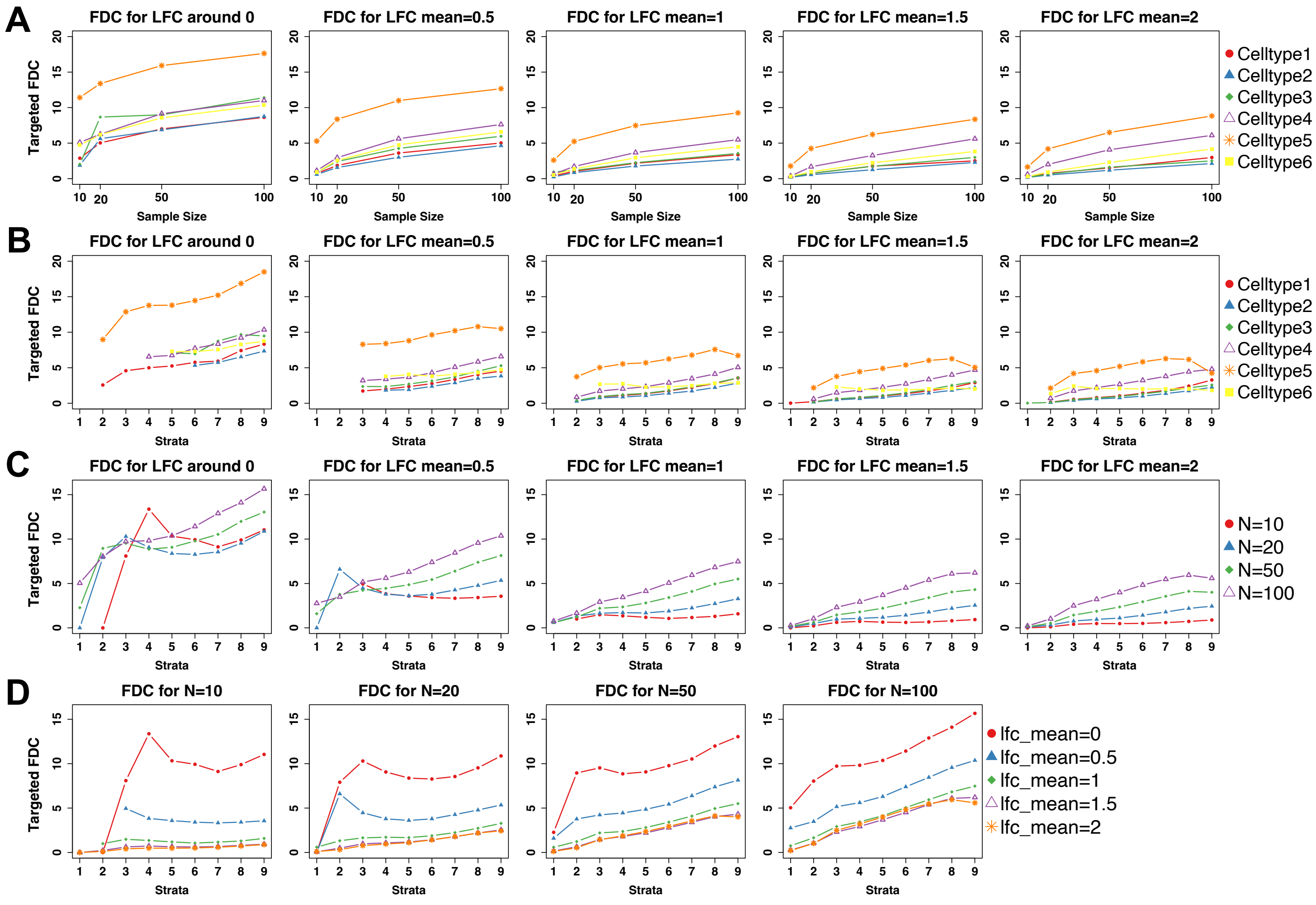

Figure S5: The targeted FDC using the threshold of 0.2 LFC as the qualifying criteria. The remaining descriptions of (A)-(D) are the same as in Figure S4.

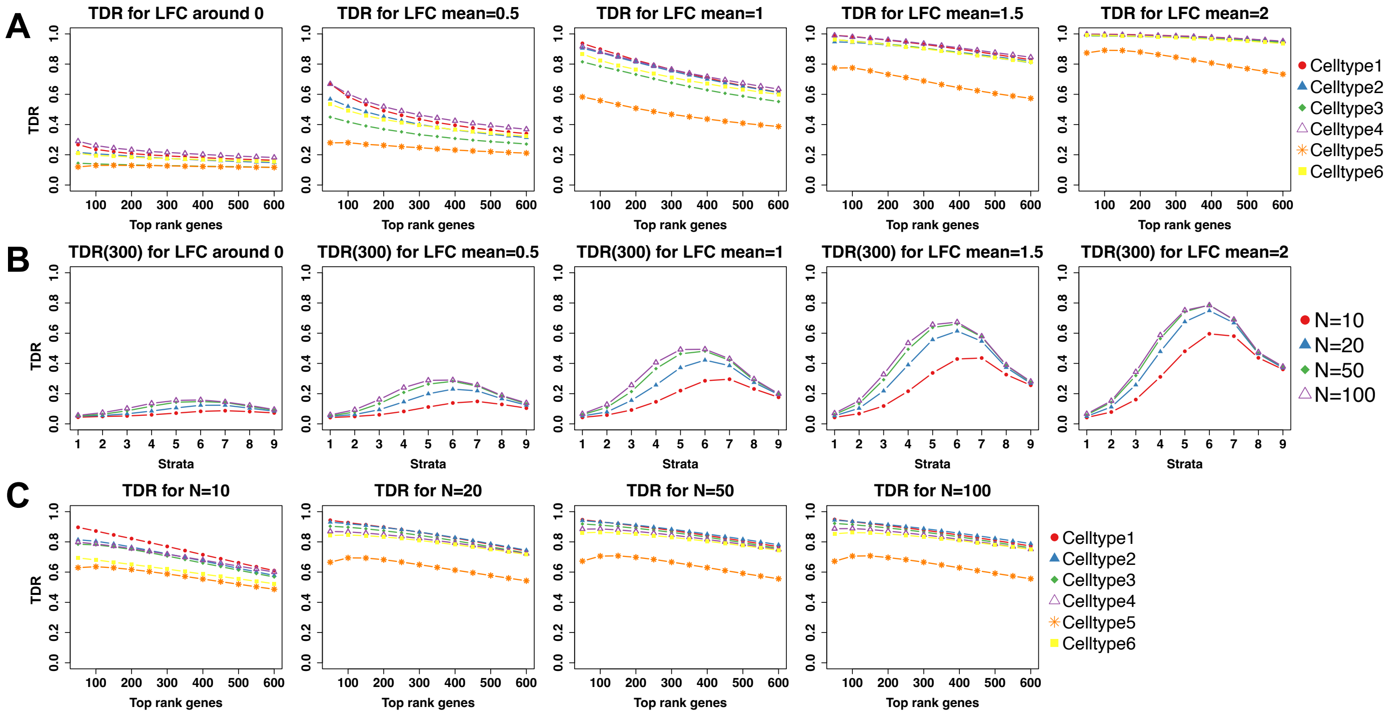

Figure S6: True discovery rate (TDR) influenced by sample size, effect size, and gene expression strata. Each point on the line plot is an average value over N=30 simulations based on a real bulk RNA-seq data with 6 cell lines (GSE60424). Log fold change (LFC) has a distribution with a mean given in each panel, with a standard deviation of 0.5. (A) TDR by top-rank genes under the scenario of increasing LFC mean value, each line represents one cell type. TDR was averaged across sample sizes. (B) TDR at the top 300 genes by strata under the scenario of increasing LFC mean value, each line representing one cell type. TDR was averaged across cell types. (C) TDR by top-rank genes under the scenario of increasing sample size, each line represents one cell type. TDR was averaged across effect sizes.

3 Impact of the number of cell types

Users can obtain results from different numbers of cell types using the `simFromData()` function in the *cypress* package. Specifically, users need to specify the total number of cell types using the `‘CT_index’` arguments and set the `‘CT_unk’` syntax to `‘True’`. Under this scenario, the *cypress* package assumes that only bulk RNA-seq data is available for users. Therefore, it will automatically run an RF deconvolution algorithm to estimate the simulation parameters for prospective usage.

We utilize bulk RNA-seq data from a large Autism Spectrum Disorder (ASD) study Gandal et al. [2018], Parikshak et al. [2016], which is attached to the *cypress* package, to evaluate how the power is affected by the number of cell types. We set `‘CT_index’` to 3, 6, 8, and 10 and `‘CT_unk’` to `‘True’` to simulate the conditions with different numbers of cell types. The simulation scenario is set with a sample size of $N = 50$ for each group, a total of $G = 3000$ genetic features, and an effect size defined as $LFC = 2$.

Table S3 below summarizes the results across different numbers of cells. A decrease in the total number of cell types can lead to increased power or decreased FDC. This conclusion aligns with previous findings that cell type proportions are positively associated with the csDE genes detection accuracies Meng et al. [2023]. The explanation is straightforward: for cell types that have small proportions, the technical noises could easily overwhelm biological signals. Thus, detecting csDE genes among minor cell types is a more challenging task.

	Number of cell types			
Results	3	6	8	10
Power	0.948	0.934	0.908	0.856
FDC	15.02	12.62	11.15	9.41

Table S3: Impacts of the total number of cell types on power and false discovery cost (FDC). The sample size for each group is set at 50, and the effect size is defined as LFC=2.

4 Impact of the number of genetic features and simulations

We conducted additional simulations at various combinations of the total number of genetic features (10,000, 20,000, and 30,000) and simulation counts (20, 30, 40, and 50). We notice that these factors have a minor impact on the power assessment, as shown in Table S4. Here, the sample size is fixed at N=50 for each group, the effect size is set to LFC=2, and the percentage of DE genes for each cell type is set at 5%.

Power	Simulations			
Genes	20	30	40	50
10k	0.891	0.890	0.890	0.890
20k	0.888	0.888	0.888	0.888
30k	0.884	0.884	0.884	0.884

Table S4: Power assessment across different combinations of genetic features and simulation counts. The sample size for each group is set at 50, the effect size is defined as LFC=2, and the percentage of DE genes for each cell type is set at 5%

5 Impact of the initial reference-based deconvolution

We additionally investigated relationship between RF deconvolution accuracy and simulation results. We conducted additional simulations using parameters estimated from GSE60424. Deconvolution accuracy is measured by the correlation between the simulated and the estimated cell type proportions. We design three levels of accuracy – low, medium, and high – by adjusting the total number of relevant genes used in the RF deconvolution process. Three levels are represented by distinct colors in the left panel of Figure S7, which displays a scatterplot of csDE gene calling sensitivity against the correlation between true and estimated cell type proportions. The results indicate that using more genes for RF deconvolution leads to higher deconvolution accuracies and subsequently enhances sensitivities. The right panel of Figure S7 presents boxplots of the sensitivities for different levels of accuracy. We recommend users who only have bulk RNA-seq data available apply a Reference-Based (RB) deconvolution approach outside of the *cypress* package. RB methods provide more precise deconvolution results, yielding more accurate simulation estimates.

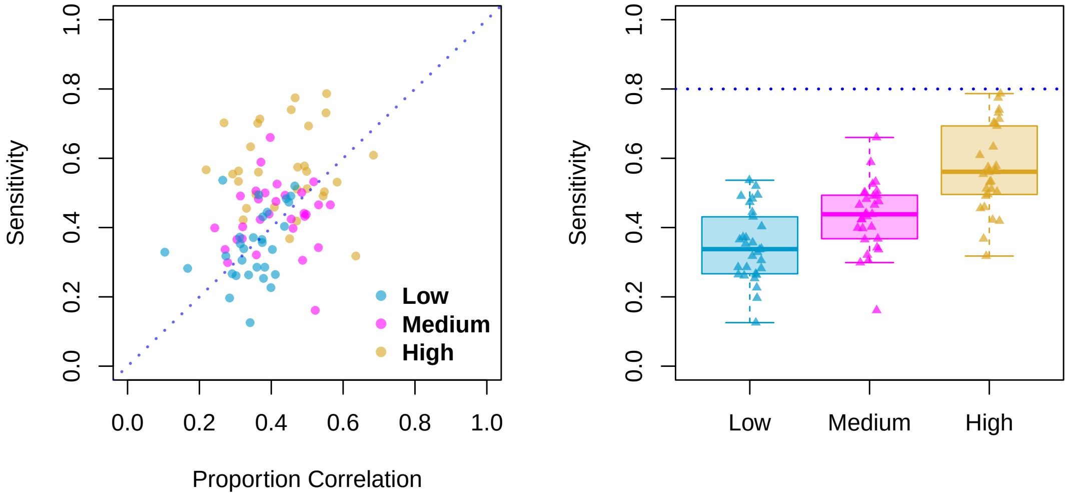

Figure S7: The left panel displays a scatter plot showing the relationship between simulated sensitivity and deconvolution accuracy, defined as the correlation between the true and estimated proportions. Different colors indicate various levels of deconvolution accuracy, determined by the total number of genetic features used in the RF deconvolution process. The right panel presents box plots of sensitivities for each level of deconvolution accuracy.

6 Impact of the estimated cell-type-specific expression matrices

To explore how the precision of estimated cell-type-specific expression matrices affects power inferences, we conducted additional simulations based on parameters estimated from GSE60424. Initially, we need to create different levels of precision for estimating cell-type-specific expression matrices, measured by the correlation between the true and estimated. This could be achieved by introducing levels of random noises (±1%,±10%,±20%,±30%) to the simulated cell type proportions. As shown in the left panel of Figure S8, the precision of the estimated cell-type-specific expression matrices decreases with increasing noise levels, indicated by different colors. When the precision of the estimated cell-type-specific expression matrices decreases, the power inference also diminishes. This trend is also demonstrated in the right panel of Figure S8, which shows power boxplots for different noise levels added to the cell proportions.

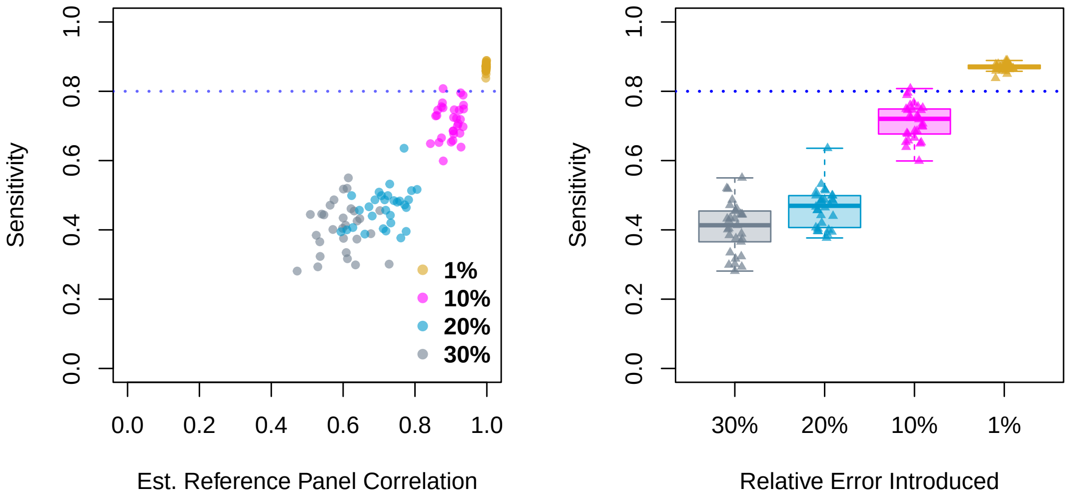

Figure S8: The left panel displays the relationship between sensitivities and precisions of the estimated cell-type-specific expression matrices, defined as the correlation between the true and estimated. This correlation is categorized by different levels of noise added to cell proportions. The right panel presents boxplots of the simulated sensitivity across different noise levels added into cell proportions.

7 Impact of DE genes proportions

cypress assumes each DE gene is present in only one cell type at a time and does not account for a gene being DE in multiple cell types. Users can specify the percentage of DE genes for each cell type, and the total number of DE genes is the sum across cell types. For example, if a user sets 5% DE genes for each of the six cell types, the total would be 30%. In Table S5, powers are evaluated under different DE genes percentages (1%, 2%, 5%, and 8% for each cell type) and effect sizes. It shows the amount of DE genes assigned to each cell type has minimal impact on power, at the current design.

Power	%DE genes for each cell type			
LFC	1%	2%	5%	8%
1	0.631	0.632	0.644	0.659
2	0.877	0.879	0.884	0.889

Table S5: Simulated power under different percentages of DE genes per cell type. The sample size for each group is set at 50, and the effect size is defined as LFC=1 and 2

8 Simulated data evaluations

cypress package contains simulation parameters derived from three distinct sources: an immune-associated disease (IAD) study (GSE60424) Linsley et al. [2014], a pediatric inflammatory bowel disease (IBD) study (GSE57945) Haberman et al. [2014], Loberman-Nachum et al. [2019], and an Autism Spectrum Disorder (ASD) study Gandal et al. [2018], Parikshak et al. [2016]. We evaluate the simulated data generated from these three sources by overlapping density plots with the respective real data, as shown in Figure S9. We can conclude that our simulation pipeline is a reliable data generation process that produces data that are highly similar to real data.

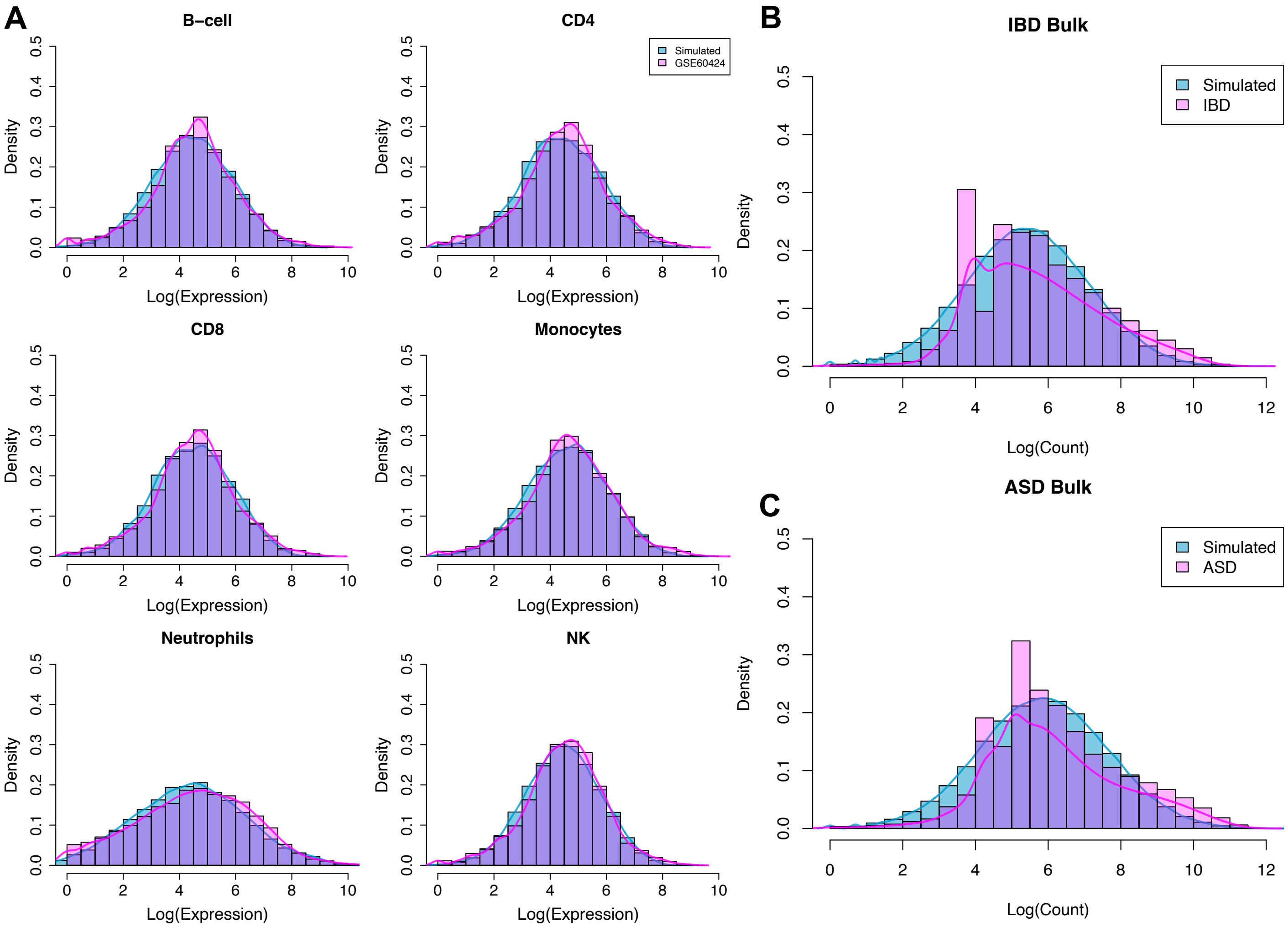

Figure S9: The distributions of the simulated RNA-seq data compared to real data. (A) Overlapped distributions of simulated cell type-specific expressions and real RNA-seq counts for each immune cell subset from GSE60424. (B-C) Comparisons of distributions between simulated datasets and real datasets, with parameters respectively estimated from IBD and ASD studies.

We additionally present a scatter plot (Figure S10) of estimated dispersion over mean for 3000 genes across 100 samples from one iteration of the data simulation process, based on the IAD data (GSE60424) simulation parameter. We observe a trend that for the genes with high mean expression, the dispersion across replicates tends to decrease accordingly.

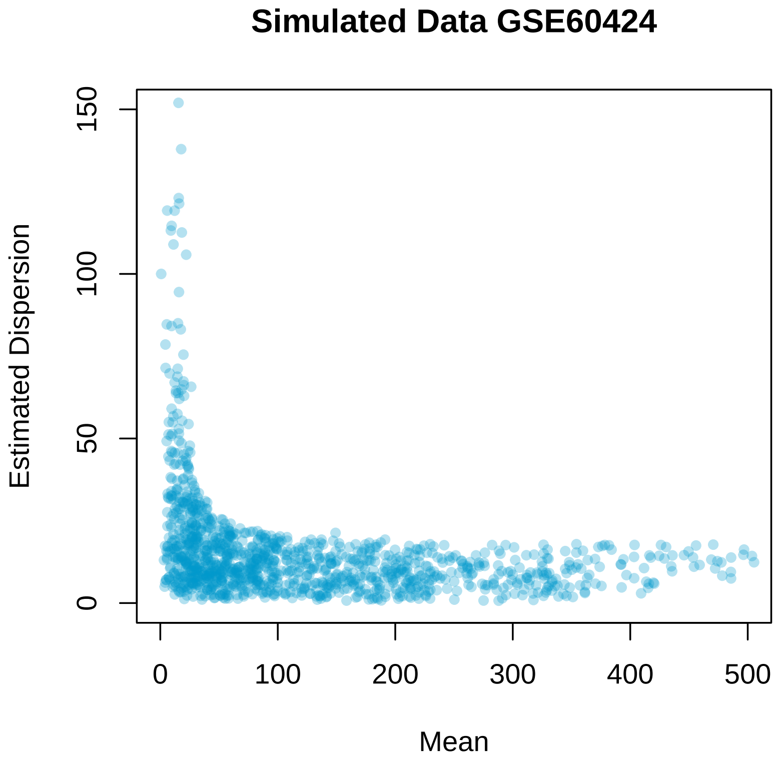

Figure S10: Dispersion estimates relative to mean for a simulated bulk RNA-seq dataset: This plot is based on simulation parameters from GSE60424. Each dot represents the dispersion estimate of an individual gene.

9 *cypress* Run time

Table S6 below shows runtime information across different numbers of genes and simulations. Increasing the number of genetic features and iterations will lead to longer run times. In general, our method is efficient in providing comprehensive evaluation results in minutes.

Run time (s)	Simulations			
Genes	20	30	40	50
10k	114.0	171.5	226.8	284.9
20k	133.1	211.7	265.5	328.8
30k	149.0	214.8	282.2	347.3

Table S6:Run time across different combinations of genetic features and simulation counts. The sample size for each group is set at 50 for each group, and the effect size is defined as LFC=2.

References

M. J. Gandal, P. Zhang, E. Hadjimichael, R. L. Walker, C. Chen, S. Liu, H. Won, H. Van Bakel, M. Varghese, Y. Wang, et al. Transcriptome-wide isoform-level dysregulation in asd, schizophrenia, and bipolar disorder. *Science*, 362(6420): eaat8127, 2018.

M. Gierliński, C. Cole, P. Schofield, N. J. Schurch, A. Sherstnev, V. Singh, N. Wrobel, K. Gharbi, G. Simpson, T. Owen-Hughes, et al. Statistical models for rna-seq data derived from a two-condition 48-replicate experiment. *Bioinformatics*, 31(22):3625–3630, 2015.

I. Gonzalez. Tutorial. statistical analysis of rna-seq data, 2014.

Y. Haberman, T. L. Tickle, P. J. Dexheimer, M.-O. Kim, D. Tang, R. Karns, R. N. Baldassano, J. D. Noe, J. Rosh, J. Markowitz, et al. Pediatric crohn disease patients exhibit specific ileal transcriptome and microbiome signature. *The Journal of clinical investigation*, 124(8):3617–3633, 2014.

E. A. Houseman, M. L. Kile, D. C. Christiani, T. A. Ince, K. T. Kelsey, and C. J. Marsit. Reference-free deconvolution of dna methylation data and mediation by cell composition effects. *BMC bioinformatics*, 17(1):1–15, 2016.

P. S. Linsley, C. Speake, E. Whalen, and D. Chaussabel. Copy number loss of the interferon gene cluster in melanomas is linked to reduced t cell infiltrate and poor patient prognosis. *PloS one*, 9(10):e109760, 2014.

N. Loberman-Nachum, K. Sosnovski, A. Di Segni, G. Efroni, T. Braun, M. BenShoshan, L. Anafi, C. Avivi, I. Barshack, D. S. Shouval, et al. Defining the celiac disease transcriptome using clinical pathology specimens reveals biologic pathways and supports diagnosis. *Scientific reports*, 9(1):16163, 2019.

G. Meng, W. Tang, E. Huang, Z. Li, and H. Feng. A comprehensive assessment of cell type-specific differential expression methods in bulk data. *Briefings in bioinformatics*, 24(1):bbac516, 2023.

N. N. Parikshak, V. Swarup, T. G. Belgard, M. Irimia, G. Ramaswami, M. J. Gandal, C. Hartl, V. Leppa, L. d. l. T. Ubieta, J. Huang, et al. Genome-wide changes in lncrna, splicing, and regional gene expression patterns in autism. *Nature*, 540(7633):423–427, 2016.

E. Rahmani, R. Schweiger, B. Rhead, L. A. Criswell, L. F. Barcellos, E. Eskin, S. Rosset, S. Sankararaman, and E. Halperin. Cell-type-specific resolution epigenetics without the need for cell sorting or single-cell biology. *Nature communications*, 10(1):3417, 2019.

A. Robitzsch. *sirt: Supplementary Item Response Theory Models*, 2022. URL `https://CRAN.R-project.org/package=sirt`. R package version 3.13-1.

H. Wu, C. Wang, and Z. Wu. Proper: comprehensive power evaluation for differential expression using rna-seq. *Bioinformatics*, 31(2):233–241, 2015.
